# Diversity in Older Adults’ Use of the Internet: Identifying Subgroups Through Latent Class Analysis

**DOI:** 10.2196/jmir.6853

**Published:** 2017-05-24

**Authors:** Leonieke C van Boekel, Sebastiaan TM Peek, Katrien G Luijkx

**Affiliations:** ^1^ Tilburg School of Social and Behavioral Sciences Department Tranzo Tilburg University Tilburg Netherlands

**Keywords:** Internet, aged, cluster analysis, health

## Abstract

**Background:**

As for all individuals, the Internet is important in the everyday life of older adults. Research on older adults’ use of the Internet has merely focused on users versus nonusers and consequences of Internet use and nonuse. Older adults are a heterogeneous group, which may implicate that their use of the Internet is diverse as well. Older adults can use the Internet for different activities, and this usage can be of influence on benefits the Internet can have for them.

**Objective:**

The aim of this paper was to describe the diversity or heterogeneity in the activities for which older adults use the Internet and determine whether diversity is related to social or health-related variables.

**Methods:**

We used data of a national representative Internet panel in the Netherlands. Panel members aged 65 years and older and who have access to and use the Internet were selected (N=1418). We conducted a latent class analysis based on the Internet activities that panel members reported to spend time on. Second, we described the identified clusters with descriptive statistics and compared the clusters using analysis of variance (ANOVA) and chi-square tests.

**Results:**

Four clusters were distinguished. Cluster 1 was labeled as the “practical users” (36.88%, n=523). These respondents mainly used the Internet for practical and financial purposes such as searching for information, comparing products, and banking. Respondents in Cluster 2, the “minimizers” (32.23%, n=457), reported lowest frequency on most Internet activities, are older (mean age 73 years), and spent the smallest time on the Internet. Cluster 3 was labeled as the “maximizers” (17.77%, n=252); these respondents used the Internet for various activities, spent most time on the Internet, and were relatively younger (mean age below 70 years). Respondents in Cluster 4, the “social users,” mainly used the Internet for social and leisure-related activities such as gaming and social network sites. The identified clusters significantly differed in age (*P*<.001, ω^2^=0.07), time spent on the Internet (*P*<.001, ω^2^=0.12), and frequency of downloading apps (*P*<.001, ω^2^=0.14), with medium to large effect sizes. Social and health-related variables were significantly different between the clusters, except social and emotional loneliness. However, effect sizes were small. The minimizers scored significantly lower on psychological well-being, instrumental activities of daily living (iADL), and experienced health compared with the practical users and maximizers.

**Conclusions:**

Older adults are a diverse group in terms of their activities on the Internet. This underlines the importance to look beyond use versus nonuse when studying older adults’ Internet use. The clusters we have identified in this study can help tailor the development and deployment of eHealth intervention to specific segments of the older population.

## Introduction

In Western societies, Internet use is widespread and is increasingly important in diverse aspects of everyday life. For instance, Internet is indispensable in communication; access to news and information; and administrative applications such as applying for allowance, tax declaration, or Internet banking. Age is known to be strongly related to the likelihood that individuals use the Internet. In the Netherlands, among adults aged 65 years and older, 77.8% have access to the Internet, compared with 94% of Internet users among the whole population [1]. In the United States, Internet use among older adults is lower compared with the Dutch older adults, namely, 64% in 2016 [2]. Compared with other European countries, Internet access and using the Internet for Internet banking and social media is higher among older adults in the Netherlands [3]. Internet use among older adults in the Netherlands has increased in the last decade, although 50% of adults aged 75 years or older have never used the Internet [1]. Knowledge and insight in the Internet use of older adults is of importance since the Internet may be related to self-management, such as instrumental activities of daily living (iADL) [4]. The Internet has the potential to enhance social capital among older adults, for instance expanding or maintaining social contacts, decreasing loneliness [5], and enlarging access to information [6]. In addition, the Internet may be used for communication with health professionals or informal care givers, which also enhances self-care.

The digital divide framework [7,8] suggests a societal gap between individuals who use and who do not use the Internet. Groups that are traditionally more disadvantaged in socioeconomic sense also appear to be at a greater likelihood to not having access to the Internet (eg, [9,10]). However, this framework focuses on a comparison of users and nonusers of the Internet, and it does not account for the diversity in use of the Internet. Conversely, the usage gap suggests that the potential benefit of the Internet may be related to the activities for which the Internet is being used. For instance, Internet activities related to information, career, or education offer Internet users more chances and resources than Internet activities which are limited to entertainment purposes (eg, [7,11,12]). Higher educated people more actively use the Internet, and they mainly use the Internet for searching information whereas lower educated people use the Internet more frequently for entrainment purposes [13]. In addition, age is also related to usage of the Internet; older adults more often use the Internet for a shorter period of time compared with younger persons and are less likely to use the Internet for activities such as email and Web-based shopping [9]. Compared with younger persons, older adults gain fewer benefits from using the Internet [14]. These findings underline the to investigate the purposes and activities older adults use the Internet for. Until now, research has merely focused on comparing older adults’ use versus nonuse of the Internet (eg, [9,15,16]), overlooking the activities and purposes for which the Internet is being used. Older adults are a heterogeneous group, and the Internet use may be very diverse among this group. Understanding the diversity of the use of Internet use among older adults provides insight in the potential benefits of using the Internet for different subgroups of older adults.

The objective of this study was to identify and describe the diversity in older adults’ activities on the Internet and whether this diversity is related to social and health-related variables. The following research questions were formulated: (1) Which subgroups or clusters can be identified among older adults based on their Internet activities? (2) What are the features of these subgroups and how do they differ in their Internet activities, time spent on the Internet, and demographic variables? (3) Is there a difference between the subgroups concerning social and emotional loneliness, psychological health, and activities of daily living (ADL) of older adults?

## Methods

### Recruitment

We used data collected by an existing Internet panel that is representative of the Dutch population, namely, longitudinal Internet studies for social sciences (LISS) panel. This panel is administered by CentERdata, a Dutch research institute specialized in data collection. Panel members receive questionnaires every month and completed questionnaires are rewarded. The panel is based on a true probability sample of households drawn from the population register by Statistics Netherlands. Households are invited to participate in the panel, and people without an appropriate computer or Internet connection are provided equipment, insuring a representative sample. The LISS panel consists of 4500 households with approximately 7000 individuals.

For this study, data from 2 different questionnaires that are annually completed by LISS panel members were combined, namely, the LISS core studies “social integration and leisure” (data collection in October and November 2015) and “health” (data collection in July and August 2015). Demographic information such as age, gender, and marital status were measured in November 2015. Panel members aged 65 years and older were selected if they completed the “social integration and leisure” questionnaire that included questions regarding Internet use (N=1608). In addition, respondents were included in the analyses when they reported to have access and use the Internet, which was the majority 88.18% (1418/1608).

### Measures

All measures were taken from annual core studies among the LISS panel members and therefore, questions were developed and tested by CentERdata. The core study, “social integration and leisure,” provided information regarding Internet use of the respondents. Web-based activities were assessed by 17 dichotomous items (never or ever spend time on this particular functionality or Web-based activity). Web-based activities included financially related activities (eg, “comparing products and searching product information,” “Internet banking”), functional and more traditional activities (“emailing,” “searching for information”), and social and leisure-related Internet activities (eg, “reading and viewing social media,” “playing Internet or Web-based games”). The amount of time spent on the Internet was asked by the following items: “Can you indicate how many hours you use the Internet on a computer or laptop/tablet/smartphone per week, on average (including emailing), besides when completing questionnaires of this panel? These items were added up to an overall amount of hours using the Internet. Answers ranged between 0 and 175 h per week. Since 175 h per week is an obvious outlier, we categorized the answers into 7 categories ranging from 1: ≤5 h, 2: 5-10 h, 3: 10-15 h...7: ≥30 h per week. Frequency of downloading apps was assessed by the items: “How often do you download apps on your tablet / smartphone” (1=never to 7=almost every day). We calculated mean scores based on both items with higher scores representing more frequent downloading apps.

Social and emotional loneliness was measured with the 6-item version of the loneliness scale of de Jong Gierveld [17]. Two examples of items are: “I experience a general sense of emptiness” and “There are many people I can trust completely” (answers: yes, no, more, or less). Overall scores for social as well as emotional loneliness were calculated ranging from 0-3, and high scores indicated more loneliness. The Mental Health Inventory 5 [18] was used to measure psychological well-being. This scale asks respondents how they felt in the last 4 weeks, for example, did you feel “calm and peaceful” or “depressed and gloomy” (6-point scale never—continuously). Sum scores were calculated in which higher scores indicated better psychological well-being [19]. ADL and iADL were measured with 2 standardized and frequently used questionnaires (eg, [20,21]) that assess limitations in general daily activities due to health problems. ADL was measured with 7 items on a 5-point scale (without any trouble—not at all): “Can you indicate, for each activity, whether you can perform it, dressing and undressing including shoes and socks / walking across the room / bathing or showering / eating, such as cutting you food into small bits / getting in and out of bed / using the toilet, including sitting down and standing up, reading a map to find your way in an unfamiliar area.” iADL was measured with 6 items on the same question and scale: preparing a hot meal / shopping telephoning / taking medicines / performing housekeeping work or maintaining the garden / taking care of financial affairs such as paving bills and keeping track of expenditure. Higher scores represent having more problems with performing iADL and ADL. Finally, demographic information that was collected included: age, gender, marital status, education level, urbanization of place of residence, and ethnicity.

### Statistical Analysis: Latent Class Analysis

The first step in the analyses was performing a latent class analysis (LCA) to identify underlying structure of the categorical data about Internet use among older people. LCA is a statistical and probabilistic method that can be used to classify individuals from a heterogeneous group into smaller more homogenous unobserved subgroups [22]. LCA was performed using the program Latent GOLD Choice 5.0 [23]. Variables included in the LCA were Web-based activities mentioned by at least 15% of the study population in order to avoid inclusion of activities that were rarely done. The Web-based activities (dichotomous yes/no) included in the LCA were (1) email; (2) searching for information; (3) searching for and comparing products or product information; (4) purchasing items; (5) Internet banking; (6) reading Web-based news and magazines; (7) reading and viewing social media (eg, Facebook, Instagram, Twitter, YouTube, LinkedIn, Google+, Pinterest, Flickr, or similar services); (8) chatting or video calling or sending messages via social media such as Instagram or Skype; (9) playing Internet games or Web-based gaming); (10) watching Web-based films or TV programs; (11) newsgroups; and (12) posting messages, photos, and short films on social media yourself (eg, Facebook, Instagram, Twitter, YouTube, LinkedIn, Google+, Pinterest, Flickr, or similar services). Model fit indices were used to select the latent model and number of clusters that is not too complex, yet also had a good fit with the data. Bayesian information criterion (BIC) and the Akaike’s information criterion 3 (AIC3) were used, that are both relative indicators of model fit. Lower values indicate better fit of the model to the data. Classification error represents the chance that a respondent is assigned to the wrong cluster and should be ideally around 10%. In case bivariate residuals were high, the assumption of local independence may be violated. Therefore, direct effects between variables were added post-hoc to the model one by one in order to reach a solution in which bivariate residuals are ≤10.

### Statistical Analysis: Describing and Comparing Identified Clusters

The second step in the analyses was describing and comparing the identified clusters. SPSS version 22 was used to conduct these analyses. A probability level of *P* ≤.01 was used. Descriptive analyses were carried out to describe the different clusters on Internet use variables and demographic variables. Chi-square test (categorical variables) and analysis of variances (ANOVAs) (continuous variables) were conducted to compare the identified clusters on Internet use variables, demographic variables, and social and emotional loneliness, psychological health, ADL, and iADL. For the ANOVA, the robust Welch *F* test was used in case the assumption of homogeneity of variances was violated. To compare the clusters pairwise, the following post-hoc tests were used: Bonferroni and Games-Howell post-hoc tests, in case assumption of homogeneity of variance was violated. Omega squared (ω^2^) was calculated as effect size estimate, as ω^2^ is robust when one of the assumptions is being violated [24]. Interpretation of ω^2^ was as follows: ω^2^≤0.06 small effect, ω^2^>0.06<0.14 medium effect, and ω^2^≥0.14 large effect.

## Results

### Description of Study Sample

In total, N=1418 respondents were included for the analyses who were individuals aged 65 years and older and using the Internet. Of these respondents, 52.82% were men (749/1418). Mean age of the respondents was 71.8 (standard deviation, SD 5.7). Of the selected sample, 8.04% (114/1418) was provided with equipment from LISS panel to be able to fill in the questionnaires monthly. Table 1 shows detailed background information of the study sample.

**Table 1:** Background data of the study sample (N=1418).

Variable		n (%)
Age (mean 71.79, SD 5.68, range 65-93)		1418 (100.00)
**Gender**		1418 (100.00)
	Men	749 (52.82)
	Women	669 (47.18)
**Marital status**		1418 (100.00)
	Married	931 (65.66)
	Separated	4 (0.28)
	Divorced	173 (12.20)
	Widow or widower	235 (16.57)
	Never been married	75 (5.29)
**Level of education^a^**		1416 (99.86)
	Low education	618 (43.64)
	Middle education	346 (24.44)
	High education	452 (31.92)
**Ethnicity**		1409 (99.37)
	Dutch background	1240 (88.01)
	First generation foreign, Western background	61 (4.33)
	First generation foreign, non-Western background	20 (1.42)
	Second generation foreign, Western background	82 (5.82)
	Second generation foreign, non-Western background	6 (0.43)
**Urbanization of place of residence**		1413 (99.65)
	Extremely urban	160 (11.32)
	Very urban	378 (26.75)
	Moderately urban	310 (21.94)
	Slightly urban	357 (25.27)
	Not urban	208 (14.72)

^a^Low education refers to primary education or prevocational secondary education. Middle education refers to preuniversity education or secondary vocational education. High education refers to higher professional education or university education.

### Results Latent Class Analysis

We compared the model fit indices, number of parameters, and classification error for models ranging from 1-8 clusters (see Table 2). The 4-cluster model was chosen as the most appropriate model since BIC (16602.92) was lowest and also AIC3 (16385.81) was low. The classification error was appropriate for the 4-cluster model (0.15). We included 4 direct effects in the model in order to decrease bivariate residuals: (1) newsgroups—reading Web-based news and magazines, (2) searching for information—email, (3) product information—searching for information, (4) reading Web-based news and magazines—watching Web-based films or TV programs. In this model with direct effects, bivariate residuals were all below 7. The entrophy R^2^ of the 4-cluster model with direct effects was 0.71.

**Table 2:** Results of the latent class analysis (N=1418).

Model	LL^a^	BIC^b^ (LL)	AIC3^c^ (LL)	# parameters	Classification error
1-cluster	−9040.46	18168.00	18116.91	12	0
2-cluster	−8380.13	16941.69	16835.27	25	0.08
3-cluster	−8182.31	16640.38	16478.61	38	0.13
4-cluster	−8116.41	16602.92	16385.81	51	0.15
5-cluster	−8076.15	16616.74	16344.29	64	0.16
6-cluster	−8040.57	16639.93	16312.14	77	0.19
7-cluster	−8014.25	16681.63	16298.50	90	0.20
4-cluster with direct effects^d^	−8039.69	16478.51	16244.37	55	0.15

^a^LL: Log likelihood.

^b^BIC: Bayesian information criterion.

^c^AIC3: Akaike’s information criterion 3.

^d^Four direct effects were included in the model based on bivariate residuals, namely (1) newsgroups—reading Web-based news and magazines, (2) searching for information—email, (3) product information—searching for information, and (4) reading Web-based news and magazines—watching Web-based films or TV programs.

### Results Describing and Comparing Identified Clusters

Cluster 1 included 36.88% (523/1418) of the respondents, and these respondents can be described as the “practical users.” The majority of respondents in this cluster used the Internet for functional and financially related activities such as “comparing products and searching product information,” “purchasing items,” and “Internet banking.” In addition, “email” and “searching for information” was a frequently mentioned activity for which these practical users used the Internet. Among the practical users, the amount of men was high compared with the other clusters (65.0%, 340/523, *P*<.001 *)* although effect size was small (Cramer *V*=.21). Cluster 2 comprised 32.23% (457/1418) of the respondents and can be labeled as the “minimizers.” Respondents in this group spent the lowest amount of hours per week on the Internet compared with the other clusters (see Tables 4 and 5) and reported the lowest frequencies of Internet activities compared with the other clusters (see Table 3). The minimizers mainly used the Internet for traditional purposes such as “email” and “searching for information” although the frequency of these activities were lowest compared with the other clusters. The mean age of the minimizers (mean 73.8, SD 6.3) was significantly higher compared with the other clusters with a medium effect size (Welch *F*=36.7, *P*<.001, ω^2^=0.07). Respondents in cluster 3 were labeled as the “maximizers” and included 17.77% (252/1418) of the respondents. The diversity of reported Internet activities in this cluster was high; and for many Internet activities, the maximizers reported the highest frequency of an Internet activity of all clusters, for instance, “watching Web-based films or TV programs,” “downloading software,” “reading Web-based news or magazines,” and “chatting, video calling, sending messages.” The mean age of the maximizers was lower compared with other clusters (mean 69.6, SD 4.4) and they spent the highest amount of hours per week on the Internet compared with the other clusters with a medium effect size (mean 3.4, SD 2.1, Welch *F*=63.3, *P*<.001, ω^2^=0.12). The maximizers downloaded apps on their devices significantly more often compared with the other clusters with a large effect size (Welch *F*=81.2, *P*<.001, ω^2^=0.14). Among the maximizers, the amount of men and women as well as different education levels is quite equally distributed. In sum, the maximizers more frequently used the Internet and used the Internet for a great diversity of Internet activities. Finally, cluster 4 comprised 13.12% (186/1418) of the respondents and can be described as the “social users.” The social users mainly used the Internet for social and leisure-related Internet activities. For instance, “reading and viewing social media,” “playing Internet or Web-based games,” and “posting messages or photos or short films on social media” was frequently mentioned as Internet activity by social users. The amount of women is high among the social users (63.98%, 119/186). The social users are most comparable with the practical users (cluster 1) in terms of age, time spent on the Internet, and frequency of downloading apps (see Tables 4 and 5). The majority of the social users, as was the case among the minimizers, were respondents with lower education (respectively 54.84%, 102/186 and 56.24% 257/457).

ANOVA tests were carried out to compare the 4 clusters on social and health-related variables, namely, social and emotional loneliness, psychological well-being, ADL, and iADL. Overall, no big differences were found in social and health-related variables between the identified clusters since effects sizes were all rather small (see Tables 4 and 5). Nevertheless, the practical users reported significant higher psychological well-being compared with the minimizers (practical users: mean 79.9, SD 13.6; minimizers: mean 76.7, SD 15.4). Additionally, in iADL, significant differences were found between the minimizers on the one hand (mean 9.2, SD 3.4) and the maximizers and practical users on the other hand (practical users: mean 8.3, SD 2.3; maximizers: mean 8.2, SD 2.1). This indicated that the minimizers had more problems with iADL compared with the practical users and the maximizers. Finally, the minimizers scored significantly lower in their experienced health compared with the maximizers (minimizers: mean 2.8, SD 0.7; maximizers: mean 3.0, SD 0.7).

**Table 3:** Frequency (%) of respondents ever spending time on an Internet activity per cluster.

Internet activity	Practical users	Minimizers	Maximizers	Social users	Chi-square *P* value	Cramer *V*
	n=523	n=457	n=252	n=186		
Email	99.6	83.2	100	98.9	<.001	.33
Searching for information	98.5	79.0	98.0	91.4	<.001	.31
Comparing products or product information	94.8	33.9	100	53.2	<.001	.64
Purchasing items	81.8	8.5	100	14.0	<.001	.79
Watching Web-based films or TV programs	15.1	4.6	38.5	17.2	<.001	.31
Downloading software or music or films^a^	15.1	2.8	28.2	10.2	<.001	.26
Internet banking	98.1	46.4	95.6	68.8	<.001	.55
Playing Internet or Web-based games	20.8	19.3	40.5	52.7	<.001	.27
Reading Web-based news or magazines	55.3	19.7	73.0	48.9	<.001	.39
Newsgroups	18.4	10.1	29.8	24.7	<.001	.18
Reading and viewing social media	23.5	8.5	99.6	93.5	<.001	.77
Reading or writing blogs^a^	7.3	1.8	21.8	14.0	<.001	.25
Posting messages or photos or short films on social media	1.0	2.4	59.9	57.5	<.001	.67
Chatting or video calling or sending messages	33.3	5.9	80.6	52.7	<.001	.55
Dating websites^a^	1.5	0.9	2.8	3.8	.05	.07
Visiting forums and communities^a^	3.3	0.9	11.9	3.8	<.001	.19
Other activities	15.1	5.9	32.1	14.0	<.001	.25

^a^Not included in the latent class analysis because frequency of activity mentioned by <15% of the respondents.

**Table 4:** Comparison (chi-square tests) of the identified clusters on demographic variables.

Demographic variables		Practical users	Minimizers	Maximizers	Social users	Χ^2^	*P* value	Cramer *V*
**Gender, n (%)**						63.4	<.001	.21
	Men	340 (65.0)	206 (45.1)	136 (54.0)	67 (36.0)			
	Women	183 (35.0)	251 (54.9)	116 (46.0)	119 (64.0)			
**Marital status, n (%)**						27.4	.007	.08
	Married	348 (66.5)	301 (65.9)	170 (67.5)	112 (60.2)			
	Separated	1 (0.2)	-	2 (0.8)	1 (0.5)			
	Divorced	64 (12.2)	37 (8.1)	39 (15.5)	33 (17.7)			
	Widow or widower	81 (15.5)	93 (20.4)	29 (11.5)	32 (17.2)			
	Never married	29 (5.6)	26 (5.7)	12 (4.8)	8 (4.3)			
**Level of education, n (%)**						90.0	<.001	.18
	Low education	187 (35.8)	257 (56.2)	72 (28.6)	102 (54.8)			
	Middle education	121 (23.1)	96 (21.0)	83 (32.9)	46 (24.7)			
	High education	215 (41.1)	104 (22.8)	96 (38.1)	37 (19.9)			

**Table 5:** Comparison (analysis of variances) of the identified clusters on age, Internet variables, and social and health-related variables.

Variables, mean (SD^a)^	Practical users^1f^	Minimizers^2f^	Maximizers^3f^	Social users^4f^	Welch *F* or *F* ratio (df^b^)	*P* value	ω^2^
Age	71.3 (5.3)^2,3^	73.8 (6.3)^1,3,4^	69.6 (4.4)^1,2,4^	71.1 (4.9)^2,3^	36.7^e^ (3610)	<.001	0.07
Amount of hours spend on Internet per week	2.4 (1.6)^2,3^	1.6 (1.3)^1,3,4^	3.4 (2.1)^1,2,4^	2.5 (1.7)^2,3^	63.3^e^ (3550)	<.001	0.12
Frequency downloading apps	1.7 (1.8)^2,3^	0.7 (1.4)^1,3,4^	2.6 (1.7)^1,2,4^	1.5 (1.8)^2,3^	87.2^e^ (3567)	<.001	0.14
Psychological well-being	79.9 (13.6)^2^	76.7 (15.4)^1^	78.7 (14.6)	76.3 (15.5)	5.0^e^ (3558)	.002	0.01
Emotional loneliness	0.5 (0.9)	0.5 (0.9)	0.5 (0.9)	0.6 (1.0)	1.7^e^ (3576)	.17	0.00
Social loneliness	1.1 (1.2)	1.0 (1.2)	1.0 (1.12)	1.0 (1.1)	0.1 (31,411)	.96	0.00
ADL^c^	6.8 (1.9)	7.1 (2.2)	6.7 (1.8)	6.8 (1. 6)	3.9^e^ (3591)	.009	0.01
iADL^d^	8.3 (2.3)^2^	9.2 (3.4)^1,3^	8.2 (2.1)^2^	8.8 (2.4)	9.5^e^ (3588)	<.001	0.02
Experienced health	2.9 (0.7)	2.8 (0.7)^3^	3.0 (0.7)^2^	2.9 (0.7)	4.0 (31,383)	.007	0.01

^a^SD: standard deviation.

^b^df: degrees of freedom.

^c^ADL: activities of daily living.

^d^iADL: instrumental activities of daily living.

^e^Welch *F* test and Games-Howell post hoc test were used since for these variables assumption of homogeneity of variances were violated.

^f^The superscript numbers 1-4 indicate significant differences (<.01) between the clusters on Bonferroni and Games-Howell posthoc test.

## Discussion

### Principal Findings

The results of this study show that older adults are a diverse group concerning their activities on the Internet. We identified 4 clusters of older adults based on the activities for which they use the Internet. First, the minimizers are the oldest respondents (mean age 74 years old) and spend the least time on the Internet. The minimizers report the lowest frequency on most of the Internet activities and mainly use the Internet for traditional purposes such as email. Second, on the other end of the spectrum are the maximizers, who are relatively young (mean age below 70 years old), spend the most time on the Internet, and are reported to spend time on almost all of the Internet activities. Among the maximizers, the amount of men and women, as well as the different education levels, are equally distributed. The third and fourth clusters are in between the minimizers and maximizers: the practical users and social users. These clusters score in between the maximizers and minimizers regarding both time spent on the Internet and their age (mean age 71 years old). The practical users, in contrast with the social users, use the Internet mainly for financial and practical matters such as searching for information, comparing products, and Internet banking. As opposed to practical users, the social users mainly use the Internet for social and leisure related activities (social media, games, etc). The amount of men is higher among the practical users and the amount of women higher among the social users.

The clusters did not differ to a large extent in social and health-related variables. However, the minimizers reported lower psychological well-being compared with the practical users, more problems with iADL in comparison with the maximizers and practical users, and lower experienced health compared with the maximizers. In sum, it appeared that the minimizers show a somewhat lower health, but this cluster also comprised the oldest respondents (mean age 74 years old). As causality between the variables is unclear, it is unknown whether age causes older adults to be only minimally active on the Internet or that a lower health status causes lower Internet activity.

It has been established that physical and mental limitations may form a barrier for older adults to use computers and the Internet [25]. In our study sample, the majority of older adults belonged to the practical users and the minimizers. It would be worthwhile to replicate this study in the upcoming years to study whether the distribution over the clusters will change. Possibly the amount of maximizers will increase considering the fact that a new generation will be more active in using the Internet. Longitudinal research may provide insight in whether older adults shift from one cluster to the other when they become older. In addition, it is of interest to further investigate the association between health (decline), age, and older adults’ Internet activities. In particular, previous research has suggested that aging processes may lead older adults to consciously or unconsciously limit the number of Web-based activities they engage in [26]. This is in line with Baltes and Baltes’ [27] concept of “selection” that describes how seniors, who are confronted with more options than their internal and external resources can handle, are forced to concentrate their energy on a subset of those options. Further research is needed to comprehend why older adults would limit or expand their number of Web-based activities.

### Comparison With Prior Work

One study [28] also investigated differences for which adults aged 65 years and older use the Internet, although only four Internet activities were included. In line with our findings it was found that the oldest respondents, aged 80 years and older, mainly use the Internet for practical purposes categorized as email or texting [28]. As mentioned before, most research has been focused on comparing older adults who use and who do not use the Internet. However, patterns of Internet use among the general population have been investigated more extensively. As mentioned earlier, previous findings have indicated that lower educated people use the Internet more often for entertainment-related purposes [13]. This was in line with our findings—among the minimizers and the social users education level was somewhat lower. One study found indications that digital literacy, that is, using the Internet and email, was related to lower iADL impairment [29]. The results of this study found more iADL impairments among the minimizers compared with the maximizers and practical users. Furthermore, we found that in general the oldest respondents, above 73 years old, spend a smaller amount of time on the Internet compared with younger respondents (between 65 and 70 years old) which was in line with prior work [9]. In the same study it was found that being older was related to being less active on the Internet [9]. One of the reasons for this is possibly the fact that older adults are from a different technological generation and therefore find it more difficult to use nowadays technological devices [30]. In this study we also found a significant age differences between the different clusters based on older adult’s activities on the Internet. Nevertheless, age differences were rather small and the study sample consisted only of adults aged 65 years and older by which a comparison with younger respondents was not part of the study. Therefore, longitudinal research and replication of this study is recommended to study whether results are equal in the upcoming years when the younger respondents (below 70 years old) become the older ones.

### Limitations

To our knowledge this is the first study that identifies clusters of older adults based on the activities for which older adults use the Internet. The large sample size strengthens the findings of this study. We strongly recommend other studies to consider using LISS panel data since the quality of the data is excellent. The Dutch population is considered to be comparable to other Western populations in terms of Internet use; therefore, we expect that the findings of this study apply to a large extent to other Western populations. Nevertheless, attention should be paid to the following limitations. Data of two surveys were combined and data collection took place on two different moments within a time span of 3 months. We are of the opinion that the variables included in this study are quite stable and are not expected to fluctuate to a large extent in a period of 3 months. The information on which the LCA was based was limited to dichotomous information whether respondents ever spend time on a particular Internet activity or not. We did not have information about the time spent on each of the Internet activities, nor did we have information about attitudes of the respondents with regard to Internet use. In addition, no information was available about support that older adults receive in using technologies which is known to be related to older adults’ use of technologies [31,32]. Finally, due to the cross-sectional design it was impossible to investigate causality between the included variables.

### Practical Implications

In the Netherlands, considerable emphasis is placed on increasing the use of eHealth, in particular among older adults and patient with chronic illnesses [33]. However, research shows that awareness of eHealth services among Dutch users of primary care is rather low and could be improved [33,34]. The identified clusters in this study can be of use in increasing the awareness and use of eHealth interventions among older adults in two ways. First, our results provide information on which channels can be employed to raise awareness of eHealth applications. Choosing the right channels to reach older adults is of importance since our results show that older adults are only on the Web a couple of hours per week. Raising awareness through social media campaigns is likely to be effective for reaching older adults belonging to the cluster social users and maximizers. In contrast, practical users may be reached by advertising campaigns on Web-based shops or product comparison websites. Minimizers may be reached by advertising in search engines, but offline methods also need to be considers for this particular cluster. Second, the identified clusters are an indication of Internet experience among older adults. This information is useful in choosing and designing effective components of eHealth interventions. For instance, practical users and minimizers have less experience in engaging in Web-based discussions than social users and maximizers. In case an eHealth intervention contains a social discussion component, it should be taken into account that particular older adults are familiar with social media interfaces whereas other are not familiar with this. Another important difference between the clusters is the fact that practical users and maximizers are more experiences in purchasing on the Web compared with the other two clusters. This can have implications for determining the range and type of payment options of eHealth services for older adults. Finally, knowledge about the amount of older adults that engage in different Internet activities may be useful for health care organizations in their marketing strategies.

### Conclusions

The findings of this study establish that older adults are a diverse group in terms of their activities on the Internet. This underlines the importance to look beyond use versus nonuse when investigating older adults’ Internet use. The heterogeneity in activities for which older adults use the Internet is widespread and is vital to consider when attempting to stimulate or facilitate Internet use among older adults. The clusters we have identified in this study can be useful in creating awareness of eHealth interventions among specific segments of the older population.

## Abbreviations

AIC3: Akaike’s Information Criterion 3
ADL: activities of daily living
ANOVA: analysis of variance
BIC: Bayesian Information Criterion
iADL: instrumental activities of daily living
ICT: information and communication technology
LCA: latent class analysis
LISS: longitudinal Internet studies for social sciences
LL: log likelihood
